# Supplementary material for: Engineering a Plant Polyketide Synthase for the Biosynthesis of Methylated Flavonoids
Source: J Agric Food Chem. 2023 Dec 18;72(1):529–39. doi: 10.1021/acs.jafc.3c06785 (PMC10786038; doi:10.1021/acs.jafc.3c06785)
Supplement: Supplementary file 1 — jf3c06785_si_001.pdf [file jf3c06785_si_001.pdf]

Supporting Information

Engineering a plant polyketide synthase for the biosynthesis of methylated flavonoids

Bo Peng[a], Lili Zhang[b], Siqi He[a], Rick Oerlemans[b], Wim J. Quax[a], Matthew R. Groves[b], Kristina Haslinger[a*]

[a]Chemical and Pharmaceutical Biology, Groningen Research Institute of Pharmacy, University of Groningen, Antonius Deusinglaan 1, 9713AV Groningen, The Netherlands

[2]XB20 Drug Design, Groningen Research Institute of Pharmacy, University of Groningen, Antonius Deusinglaan 1, 9713AV Groningen, The Netherlands

*Email:k.haslinger@rug.nl

Contents

Supporting Materials 2

Supporting Tables 4

Supporting Figures 8

Supporting Materials

Sequences of synthetic genes

Pc4CL (*Petroselinum crispum*, GenBank accession number KX671122.1):

ATGGGTGACTGCGTTGCCCCGAAAGAGGATCTGATCTTCCGCAGCAAACTGCCGGACATTTACATTCCAAAGCATCTGCCGCTGCATACC
TATTGTTTTGAGAACATCAGCAAGGTTGGCGACAAGAGCTGTCTGATCAACGGCGCAACCGGCGAAACCTTTACCTACAGCCAGGTTGA
GCTGCTGTCCCGTAAAGTTGCCAGCGGCCTGAACAAGCTGGGCATTCAACAAGGTGATACCATTATGCTGCTGCTGCCGAACTCCCCGG
AGTACTTTTTCGCTTTCCTGGGTGCGAGCTATCGCGGTGCAATCAGCACTATGGCGAACCCATTCTTTACCAGCGCAGAAGTGATCAAGC
AACTGAAAGCGAGCCAAGCGAAGCTGATTATCACCCAGGCATGCTATGTTGACAAGGTTAAGGACTACGCAGCGGAGAAAAACATCCA
GATCATTTGTATTGACGATGCACCGCAGGATTGCCTGCACTTTAGCAAGCTGATGGAAGCGGATGAGAGCGAAATGCCGGAAGTGGTT
ATTAACAGCGATGATGTGGTGGCACTGCCGTACAGCTCTGGCACCACCGGCCTGCCGAAAGGCGTTATGCTGACCCACAAGGGTCTGGT
TACCAGCGTTGCACAACAGGTGGATGGTGATAACCCGAACCTGTATATGCACTCCGAGGATGTTATGATCTGCATCCTGCCACTGTTCCA
TATCTATAGCCTGAACGCTGTTCTGTGTTGTGGTCTGCGTGCGGGCGTTACCATTCTGATCATGCAAAAGTTCGACATTGTGCCGTTTCTG
GAGCTGATTCAGAAGTATAAGGTTACCATTGGTCCGTTTGTTCCGCCGATCGTGCTGGCCATCGCGAAAAGCCCGGTTGTTGACAAGTA
CGACCTGTCTAGCGTGCGCACCGTTATGAGCGGTGCAGCGCCGCTGGGTAAAGAGCTGGAGGACGCTGTTCGTGCGAAATTCCCGAAC
GCGAAGCTGGGTCAAGGCTATGGCATGACCGAAGCCGGTCCGGTTCTGGCGATGTGTCTGGCGTTCGCCAAAGAGCCGTATGAGATTA
AGTCTGGCGCATGCGGTACCGTTGTGCGTAACGCCGAGATGAAAATCGTTGACCCAGAAACCAACGCGTCTCTGCCGCGTAACCAGCGT
GGTGAGATTTGCATCCGTGGTGATCAGATTATGAAAGGTTACCTGAACGACCCGGAAAGCACCCGCACCACCATCGACGAAGAGGGTT
GGCTGCACACCGGTGACATTGGTTTCATCGACGATGACGATGAACTGTTCATTGTTGATCGTCTGAAAGAAATCATTAAGTACAAAGGTT
TTCAAGTTGCTCCGGCGGAGCTGGAAGCACTGCTGCTGACCCACCCGACCATCAGCGATGCCGCGGTGGTTCCGATGATTGACGAGAA
AGCGGGTGAAGTGCCAGTGGCGTTTGTTGTGCGTACCAACGGTTTTACCACCACCGAAGAAGAAATCAAACAATTTGTGAGCAAACAG
GTTGTGTTCTACAAACGTATCTTCCGCGTTTTCTTCGTTGACGCTATTCCGAAATCCCCGAGCGGCAAGATTCTGCGTAAGGATCTGCGC
GCTCGTATTGCGAGCGGCGACCTGCCGAAGTAA

Os4CL (*Oryza sativa*, GenBank accession number NP_001396278):

ATGGGGTCAGTTGCAGCCGAGGAAGTAGTCGTCTTCCGCTCTAAGCTGCCGGATATCGAAATCGATAACTCTATGACCCTGCAAGAATA
CTGCTTTGCACGTATGGCAGAGGTGGGAGCCCGCCCATGTCTTATCGACGGGCAAACTGGAGAGTCATACACTTATGCTGAAGTAGAAT
CGGCCTCGCGTCGCGCAGCCGCGGGACTTCGTCGCATGGGAGTGGGCAAGGGAGACGTAGTGATGTCACTTTTGCGCAATTGCCCAGA
GTTTGCCTTTTCTTTTCTGGGGGCTGCCCGCTTAGGAGCGGCTACTACGACTGCAAATCCATTTTATACACCCCATGAGGTACATCGCCAG
GCTGAGGCAGCGGGGGCACGCGTTATTGTGACGGAAGCCTGCGCAGTTGAAAAGGTGCGCGAGTTCGCAGCTGAACGTGGTGTGCCT
GTTGTGACTGTCGACGGTGCCTTTGATGGGTGCGTAGAATTTCGTGAAGTGCTTGCAGCGGAGGAGTTAGATGCTGACGCTGATGTCCA
CCCCGATGATGTAGTGGCGCTTCCTTATTCTTCGGGTACCACAGGCTTACCTAAGGGCGTCATGCTGACACATCGTTCCCTTATTACATCG
GTTGCGCAGCAAGTAGATGGGGAAAACCCTAATCTTTATTTCTCCAAAGATGACGTGATTTTATGCCTGCTGCCTCTTTTTCATATTTATT
CGTTAAACAGCGTTCTGCTGGCAGGGCTGCGCGCTGGTTCTACAATTGTCATCATGCGCAAATTTGATTTGGGGGCGCTTGTTGATCTGG
TCCGTAAGCATAACATTACGATTGCACCATTTGTACCACCCATCGTTGTAGAAATTGCTAAATCACCCCGCGTAACAGCTGAGGATCTGG
CCTCTATTCGTATGGTCATGTCAGGTGCAGCTCCTATGGGTAAGGATTTGCAGGACGCCTTTATGGCGAAAATCCCTAATGCAGTTTTAG
GTCAAGGGTATGGTATGACTGAGGCAGGCCCTGTTCTGGCAATGTGTCTGGCCTTCGCCAAAGAGCCGTTTAAGGTAAAGTCCGGAAG
CTGTGGTACCGTGGTTCGCAACGCTGAACTTAAGATCGTAGATCCCGACACGGGTACGTCGCTGGGTCGCAATCAATCTGGTGAGATCT
GCATCCGTGGTGAGCAGATTATGAAGGGCTACTTAAACGATCCAGAGGCTACAAAGAACACCATCGATGAGGATGGGTGGCTGCATAC
CGGAGACATTGGTTTCGTGGACGATGATGATGAGATTTTTATTGTCGACCGTCTTAAAGAAATTATCAAGTACAAGGGGTTCCAGGTAC
CACCCGCTGAATTGGAGGCTCTTCTTATCACTCACCCAGAGATCAAGGACGCGGCGGTAGTCAGCATGAAGGACGATTTGGCGGGAGA
GGTCCCTGTAGCCTTCATTGTCCGCACTGAGGGGAGCGAAATTACAGAGGACGAAATTAAAAAGTTCGTTGCCAAAGAGGTGGTGTTCT
ACAAGCGTATTAACAAAGTGTTCTTTACAGATTCCATCCCTAAAAACCCTTCCGGTAAGATCTTGCGCAAGGATCTTCGTGCTCGTCTGGC
GGCGGGTATTCCGGACGCTGTCGCTGCGGCAGCTGCAGATGCTCCTAAAAGTAGCTAA

HvCHS (*Hordeum vulgare*, GenBank accession number XP_044963194):

ATGGCAGCGGTGCGTTTGAAGGAGGTGCGCATGGCGCAGCGCGCCGAGGGTTTAGCTACAGTGCTGGCGATCGGAACGGCTGTACCT
GCAAATTGTGTTTACCAAGCGACATATCCTGACTACTACTTTCGTGTTACTAAGTCAGAGCATTTAGCCGATCTTAAAGAAAAGTTTCAGC
GCATGTGTGACAAGAGTATGATCCGCAAACGCCACATGCACCTTACGGAAGAGATTTTAATTAAGAACCCCAAAATTTGCGCCCACATG
GAGACTTCACTTGATGCCCGTCATGCCATTGCCTTGGTGGAAGTCCCGAAATTGGGCCAAGGGGCCGCCGAAAAAGCCATTAAAGAAT
GGGGCCAACCGCTTAGTAAGATCACGCACTTGGTCTTTTGCACAACATCAGGAGTTGATATGCCCGGGGCGGATTACCAGTTAACGAAG
CTCCTGGGATTGAGCCCTACTGTCAAACGCCTTATGATGTACCAACAAGGATGTTTTGGCGGAGCTACTGTATTACGCCTGGCCAAAGAT
ATTGCCGAGAACAATCGCGGGGCTCGCGTTTTAGTAGTTTGCTCGGAGATTACCGCAATGGCGTTCCGTGGCCCGTGCAAATCCCATTT
GGATTCGTTAGTAGGTCACGCATTATTCGGCGATGGAGCCGCTGCTGCAATCATCGGAGCCGATCCCGACCAATTAGACGAGCAGCCG
GTATTTCAATTGGTATCGGCGAGTCAGACAATCCTGCCAGAATCGGAGGGTGCGATCGATGGACACTTGACGGAGGCGGGCTTAACGA
TTCACCTGCTGAAAGATGTGCCGGGCTTGATCTCTGAAAACATCGAGCAGGCTCTTGAGGATGCGTTTGAGCCTTTGGGTATTCACAATT
GGAACTCTATTTTTTGGATCGCTCATCCGGGCGGGCCTGCAATTTTAGACCGCGTCGAAGATCGCGTTGGATTAGATAAAAAACGTATG
CGTGCTTCACGCGAGGTCCTTAGCGAATACGGCAACATGTCTTCAGCCTCTGTCTTATTCGTGCTTGACGTTATGCGCAAGTCGAGTGCA
AAGGATGGGTTGGCGACGACGGGCGAGGGGAAAGATTGGGGGGTGCTGTTCGGATTTGGCCCAGGACTGACCGTCGAAACCCTGGTA
TTACACTCTGTTCCTGTTCCCGTTCCCACTGCGGCTTCTGCTTAA

PhCHS (*Petunia hybrida*, GenBank accession number KP284563.1):

ATGGTGACCGTGGAAGAATACCGTAAGGCGCAACGTGCGGAAGGCCCGGCGACCGTGATGGCGATTGGCACCGCGACCCCGAGCAAC
TGCGTTGACCAGAGCACCTACCCGGATTTCTATTTTCGTATTACCAACAGCGAGCACAAAACCGACCTGAAGGAAAAATTCAAGCGTAT
GTGCGAGAAGAGCATGATTAAGAAACGTTACATGCACCTGACCGAGGAAATCCTGAAAGAGAACCCGAGCATGTGCGAATATATGGCG
CCGAGCCTGGACGCGCGTCAGGATATCGTGGTTGTGGAAGTGCCGAAACTGGGCAAAGAGGCGGCGCAGAAAGCGATTAAGGAATG
GGGTCAACCGAAAAGCAAGATCACCCACCTGGTTTTCTGCACCACCAGCGGCGTGGACATGCCGGGTTGCGATTACCAACTGACCAAAC
TGCTGGGCCTGCGTCCGAGCGTTAAGCGTCTGATGATGTATCAGCAAGGTTGCTTTGCGGGTGGCACCGTGCTGCGTCTGGCGAAAGA
TCTGGCGGAAAACAACAAGGGTGCGCGTGTTCTGGTTGTGTGCAGCGAGATTACCGCGGTGACCTTCCGTGGCCCGAACGACACCCAC
CTGGATAGCCTGGTTGGTCAGGCGCTGTTTGGTGATGGTGCGGGTGCGATCATTATCGGCAGCGATCCGATTCCGGGTGTTGAGCGTCC
GCTGTTCGAACTGGTGAGCGCGGCGCAAACCCTGCTGCCGGACAGCCATGGTGCGATTGATGGTCACCTGCGTGAAGTTGGCCTGACC
TTTCACCTGCTGAAAGACGTGCCGGGTCTGATTAGCAAAAACATCGAGAAGAGCCTGGAGGAAGCGTTCAAGCCGCTGGGCATTAGCG
ACTGGAACAGCCTGTTTTGGATTGCGCACCCGGGTGGCCCGGCGATTCTGGATCAAGTTGAAATCAAACTGGGCCTGAAGCCGGAGAA
ACTGAAGGCGACCCGTAACGTTCTGAGCAACTACGGTAACATGAGCAGCGCGTGCGTGCTGTTTATCCTGGATGAAATGCGTAAAGCG
AGCGCGAAAGAGGGTCTGGGTACCACCGGCGAGGGTCTGGAATGGGGTGTGCTGTTCGGCTTTGGTCCGGGCCTGACCGTGGAAACC
GTTGTTCTGCATAGCGTTGCGACCTAA

MsCHI (*Medicago sativa*, GenBank accession number P28012):

ATGGCGGCGAGCATTACCGCGATTACCGTGGAAAATCTGGAATATCCGGCGGTTGTGACCAGCCCGGTGACCGGCAAAAGCTACTTCCT
GGGTGGCGCGGGCGAGCGTGGCCTGACCATCGAAGGCAACTTCATTAAATTTACCGCGATCGGTGTGTACCTGGAGGACATTGCGGTT
GCGAGCCTGGCGGCGAAGTGGAAAGGCAAGAGCAGCGAGGAACTGCTGGAAACCCTGGACTTCTATCGTGATATCATTAGCGGTCCGT
TTGAAAAACTGATTCGTGGCAGCAAGATCCGTGAGCTGAGCGGTCCGGAATACAGCCGTAAAGTGATGGAGAACTGCGTTGCGCACCT
GAAGAGCGTGGGTACCTATGGCGATGCGGAGGCGGAAGCGATGCAGAAATTCGCGGAAGCGTTTAAACCGGTGAACTTCCCGCCGGG
TGCGAGCGTGTTCTACCGTCAAAGCCCGAACGGTATTCTGGGCCTGAGCTTCAGCCCGGACACCAGCATTCCGGAGAAAGAAGCGGCG
CTGATCGAAAACAAGGCGGTGAGCAGCGCGGTTCTGGAAACCATGATCGGTGAACACGCGGTTAGCCCGGATCTGAAGCGTTGCCTGG
CGGCGCGTCTGCCGGCGCTGCTGAATGAGGGTGCGTTCAAGATTGGTAACTAA

Supporting Tables

Table S1. List of plasmids and bacterial strains used in this study.

Strain	Relevant Genotype	Reference
E. coli BL21(DE3)	F - ompT hsdSB(rB -mB -) gal dem (λIts857 indI Sam7 nin5 lavUV5-T7gene1)	Invitrogen
E. coli DH5a	supE44 Δ(lacZYA-argF)U196 (Φ80ΔlacZM15) hsdR17 recA1 endA1 gyrA96 thi-1 relA1	Invitrogen
E. coli K-12 MG1655 (DE3)	K-12 F^- λ^- *ilvG*$^-$ *rfb-50 rph-1*	Nielsen *et al.*[25]

Identifier	Construct name	Backbone	Enzyme encoded		Source
			MCS1	MCS2	
c1	pETDuet::PhCHS_Pc4CL	pETduet-1	PhCHS	Pc4CL	This study
c2	pCDFDuet::_MsCHI	pCDFduet-1	MsCHI	/	This study
c3	pETDuet::HvCHS_Os4CL	pETduet-1	HvCHS	Os4CL	This study
c4	pETDuet::PhCHS_Pc4CL(del V342)	pETduet-1	PhCHS	Pc4CL (del V342)	This study
c5	pETDuet::HvCHS (A228S, D231I, Q232P, L233G, D234V)_Os4CL	pETduet-1	HvCHS (A228S, D231I, Q232P, L233G, D234V)	Os4CL	This study
c6	pETDuet::PhCHS_Pc4CL(Q214A)	pETduet-1	PhCHS	Pc4CL (Q214A)	This study
c7	pETDuet::HvCHS_Os4CL (del V340)	pETduet-1	HvCHS	Os4CL (del V340)	This study
c8	pETDuet::HvCHS_Os4CL(Q212A)	pETduet-1	HvCHS	Os4CL (Q212A)	This study
c9	pETDuet::HvCHS_Os4CL(S242A)	pETduet-1	HvCHS	Os4CL (S242A)	This study
c10	pETDuet::HvCHS(A199T)_Os4CL	pETduet-1	HvCHS (A199T)	Os4CL	This study
c11	pETDuet::HvCHS(I267F)_Os4CL	pETduet-1	HvCHS (I267F)	Os4CL	This study
c12	pETDuet::PhCHS(T197A)_Pc4CL	pETduet-1	PhCHS (T197A)	Pc4CL	This study
c13	pETDuet::HvCHS(A228S)_Os4CL	pETduet-1	HvCHS (A228S)	Os4CL	This study
c14	pETDuet::HvCHS(D231I)_Os4CL	pETduet-1	HvCHS (D231I)	Os4CL	This study
c15	pETDuet::HvCHS(Q232P)_Os4CL	pETduet-1	HvCHS (Q232P)	Os4CL	This study
c16	pETDuet::HvCHS(L233G)_Os4CL	pETduet-1	HvCHS (L233G)	Os4CL	This study
c17	pETDuet::HvCHS(D234V)_Os4CL	pETduet-1	HvCHS (D234V)	Os4CL	This study
c18	pETDuet::HvCHS(Q232P, D234V)_Os4CL	pETduet-1	HvCHS (Q232P, D234V)	Os4CL	This study
c19	pETDuet::HvCHS(Q232P, D234V)_MsCHI	pETduet-1	HvCHS (Q232P, D234V)	MsCHI	This study
c20	pCDFDuet-1::Os4CL	pCDFDuet-1	Os4CL	/	This study
c21	pET28a::HvCHS	pET28a	HvCHS	/	This study
c22	pET28a::HvCHS(Q232P, D234V)	pET28a	HvCHS (Q232P, D234V)	/	This study
c23	pET28s::Os4CL	pET28a	Os4CL	/	This study
c24	pETDuet::PhCHS_Pc4CL(Q214A, del V342)	pETduet-1	PhCHS	Pc4CL(Q214A, del V342)	This study
c25	pETDuet::HvCHS_Os4CL(Q212A, S242A, del V340)	pETduet-1	HvCHS	Os4CL(Q212A, S242A, del V340)	This study

* MCS1 and 2, multiple cloning site (each with its own T7 promoter and terminator); PhCHS, CHS from *Petunia hybrida*; MsCHI, CHI from *Medicago sativa*; Pc4CL, 4CL from *Petroselinum crispum*; HvCHS, CHS from *Hordeum vulgare*; Os4CL, 4CL from *Oryza sativa*; codon optimized for expression in *E. coli.*

Table S2. List of *E. coli* MG1655 (DE3) strains used in fermentation experiments.

Identifier	Construct name	Enzymes expressed	Source
s1	c1, c2	PhCHS, Pc4CL, and MsCHI	This study
s2	c2, c3	HvCHS, Os4CL, and MsCHI	This study
s3	c2, c4	PhCHS, Pc4CL (del V342), and MsCHI	This study
s4	c2, c5	HvCHS (A228S, D231I, Q232P, L233G, D234V), Os4CL, and MsCHI	This study
s5	c2, c6	PhCHS, Pc4CL (Q214A), and MsCHI	This study
s6	c2, c7	HvCHS, Os4CL (del V340), and MsCHI	This study
s7	c2, c8	HvCHS, Os4CL (Q212A), and MsCHI	This study
s8	c2, c9	HvCHS, Os4CL (S242A), and MsCHI	This study
s9	c2, c10	HvCHS (A199T), Os4CL, and MsCHI	This study
s10	c2, c11	HvCHS (I267F), Os4CL, and MsCHI	This study
s11	c2, c12	PhCHS (T197A), Pc4CL, and MsCHI	This study
s12	c2, c13	HvCHS (A228S), Os4CL, and MsCHI	This study
s13	c2, c14	HvCHS (D231I), Os4CL, and MsCHI	This study
s14	c2, c15	HvCHS (Q232P), Os4CL, and MsCHI	This study
s15	c2, c16	HvCHS (L233G), Os4CL, and MsCHI	This study
s16	c2, c17	HvCHS (D234V), Os4CL, and MsCHI	This study
s17	c2, c19	HvCHS (Q232P, D234V), Os4CL, and MsCHI	This study
s18	c19, c20	HvCHS (Q232P, D234V), MsCHI, and Os4CL	This study
s19	c2, c24	PhCHS, Pc4CL (Q214A, del V342), and MsCHI	This study
s20	c2, c25	HvCHS,_Os4CL (Q212A, S242A, del V340), and MsCHI	This study

Table S3. Primers used in this study.

**Primers**	**Templates**	**Primer sequences (5′ to 3′, mutant site underlined)**	**Products**
Pc4CL del V342 FP<br>Pc4CL RP	c1	AAGCCGGTCCGCTGGCGATGTG<br>GTGGTGCGGGTGCTTTCCGG	c6
Pc4CL Q214A FP<br>Pc4CL RP	c1	CAGCGTTGCACAAGCAGTGGATGGTGAT<br>GTGGTGCGGGTGCTTTCCGG	c8
HvCHS(A228S, D231I, Q232P, L233G, D234V) FP<br><br>HvCHS (A228S, D231I, Q232P, L233G, D234V) RP	c3	[PHO]GGGTGTTGAGCAGCCGGTATTTCAATTGGTATCGGCGAGTC<br>[PHO]GGGATGGGATCGGATCCGATGATTGCAGCAGC	c7
Os4CL del V340 FP<br>Os4CL del V340 RP	c3	CTGAGGCAGGCCCTCTGGCAATGTGTCT<br>AGACACATTGCCAGAGGGCCTGCCTCAG	c9
Os4CL Q212A FP<br>Os4CL Q212A RP	c3	ATTACATCGGTTGCGCAGGCGGTAGATGGGGAAAACCC<br>GGGTTTTCCCCATCTACCGCCTGCGCAACCGATGTAAT	c10
Os4CL S242A FP<br>Os4CL S242A RP	c3	ATTTATTCGTTAAACGCGGTTCTGCTGGCAGGG<br>CCCTGCCAGCAGAACCGCGTTTAACGAATAAAT	c11
HvCHS A199T FP<br>HvCHS A199T RP	c3	TTACCGCAATGACCTTCCGTGGCCCGT<br>GCCACGGAACGCCATGGTGGTAATCTCCGAGCA	c12
HvCHS I265F FP<br>HvCHS I265F RP	c3	GAGGCGGGCTTAACGTTTCACCTGCTGAAAG<br>CTTTCAGCAGGTGAAACGTTAAGCCCGCCTC	c13
PhCHS T197A FP<br>PhCHS T197A RP	c1	GATTACCGCGGTGGCCTTCCGTGGCCC<br>GGGCCACGGAAGGCCACCGCGGTAATC	c14
HvCHS A228S FP<br>HvCHS A228S RP	c3	GCAATCATCGGAtCCGATCCCGACC<br>GAGTTCCAATTGTGAATACCCAAAGGCTCAAACGC	c15
HvCHS D231I FP<br>HvCHS D231I RP	c3	CGGAGCCGATCCCATCCAATTAGACGAG<br>GAGTTCCAATTGTGAATACCCAAAGGCTCAAACGC	c16
HvCHS Q232P FP<br>HvCHS Q232P RP	c3	GCCGATCCCGACCCGTTAGACGAG<br>GAGTTCCAATTGTGAATACCCAAAGGCTCAAACGC	c17
HvCHS L233G FP<br>HvCHS L233G RP	c3	GATCCCGACCAAGGTGACGAGCAGC<br>GAGTTCCAATTGTGAATACCCAAAGGCTCAAACGC	c18
HvCHS D234V FP<br>HvCHS D234V RP	c3	CCCGACCAATTAGTTGAGCAGCCGG<br>GAGTTCCAATTGTGAATACCCAAAGGCTCAAACGC	c19
HvCHS Q232P_D234V_FP<br>HvCHS Q232P_D234V_RP	c15	CCCGACCAATTAGTTGAGCAGCCGG<br><br>GAGTTCCAATTGTGAATACCCAAAGGCTCAAACGC	c20

*5′ [PHO] means 5' phosphorylation of primers

Table S4. Apparent Michaelis-Menten kinetic parameters for turnover of feruloyl-CoA by HvCHS wildtype and HvCHS (Q232P, D234V) at a fixed concentration of malonyl-CoA of 300 µM.

**Enzyme**	**HvCHS**	**HvCHS (Q232P, D234V)**
**Best-fit values**		
Et, µM	0.05	0.05
k_{cat}, min^{-1}	2.40	2.46
K_m, µM	4.18	2.63
V_{max}, µM/min	0.12	0.12
**95% CI (profile likelihood)**		
k_{cat}, min-1	2.16 to 2.72	2.24 to 2.72
K_m, µM	3.06 to 5.60	1.99 to 3.40
**Goodness of Fit**		
Degrees of Freedom	16	16
R squared	0.9195	0.9054
Sum of Squares	0.001094	0.0009365
Sy.x	0.008271	0.007651

Table S5. Structure alignment of the new HvCHS structure (8B32) with CHS structures in the PDB with the Dali server.

**rank**	**PDB- chain**	**Z score**	**rmsd**	**lali**	**nres**	**%id**	**enzyme name with ligand**	**donor species**
1	4yjy-A	68.2	0.5	387	393	79	Chalcone Synthase 1	*Oryza sativa*
2	4wum-C	68.2	0.4	386	389	73	Chalcone Synthase	*Freesia hybrida*
7	1cgk-A	67.7	0.5	386	387	72	Chalcone Synthase 2 with naringenin	*Medicago sativa*
8	7bur-A	67.7	0.5	385	388	74	Chalcone Synthase 1	*Gkycine max* (L.)
9	1bi5-A	67.6	0.5	386	389	72	Chalcone Synthase 2	*Medicago sativa*
10	1i86-A	67.6	0.5	386	389	72	Chalcone Synthase 2 G256A	*Medicago sativa*
11	1cgz-A	67.6	0.5	386	387	72	Chalcone Synthase 2 with resveratrol	*Medicago sativa*
12	1d6f-A	67.6	0.5	386	389	72	Chalcone Synthase 2 C164A	*Medicago sativa*
13	1bq6-A	67.6	0.5	386	388	72	Chalcone Synthase 2 with CoA	*Medicago sativa*
14	1cml-A	67.5	0.6	386	389	72	Chalcone Synthase 2 with malonyl-CoA	*Medicago sativa*

Supporting Figures

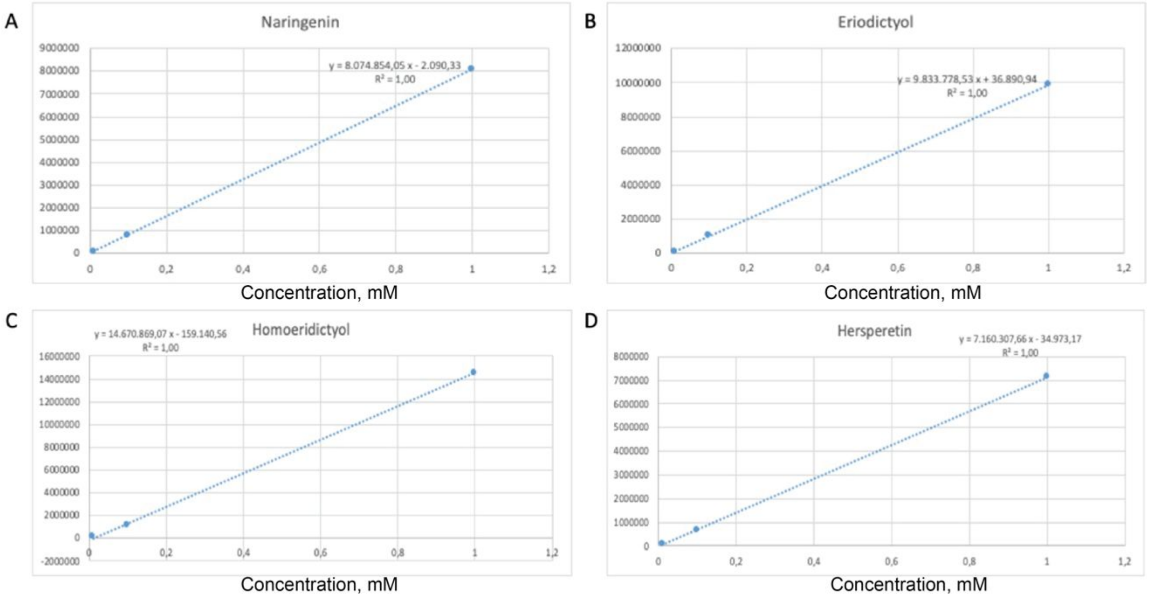

Figure S1. Calibration plot of flavonoids (A: naringenin, B: eriodictyol, C: homoeriodictyol, and D: hesperetin) dissolved in DMSO and analyzed by HPLC. The compounds were detected at 288 nm and the analysis was performed as described in the method section. The range of calibration curve was 0.01 mM to 1 mM.

A

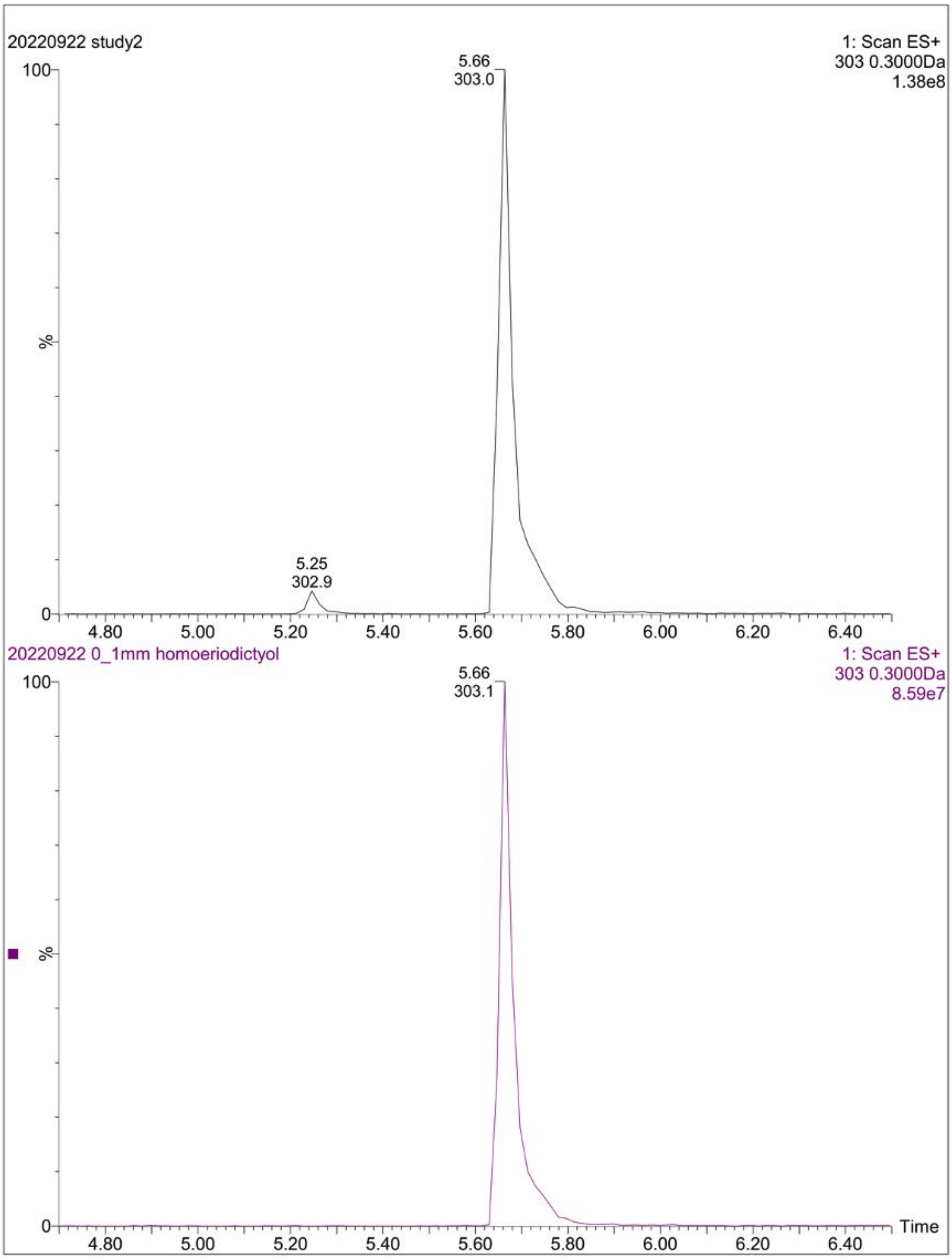
20220922 study2
1: Scan ES+
303 0.3000Da
1.38e8
5.66
303.0
5.25
302.9
100
%
0
4.80
5.00
5.20
5.40
5.60
5.80
6.00
6.20
6.40
20220922 0_1mm homoeriodictyol
1: Scan ES+
303 0.3000Da
8.59e7
5.66
303.1
Time

B

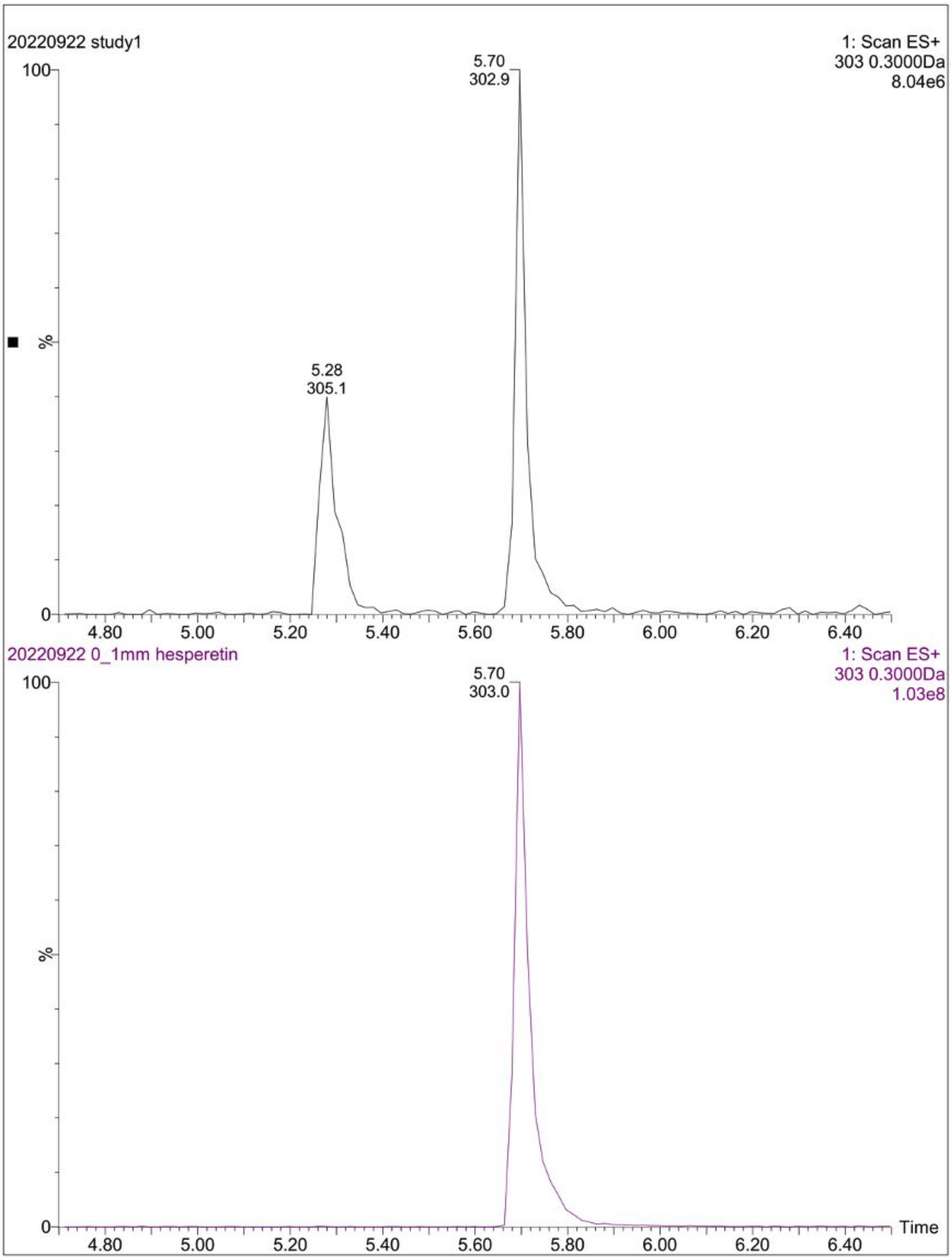

Figure S2. Extracted ion chromatograms at *m/z 303* [M+H+] in positive ion channel of the fermentation products with strain s2 expressing HvCHS wild type (black) and the analytical standards (pink). (A) homoeriodictyol (B) hesperetin.

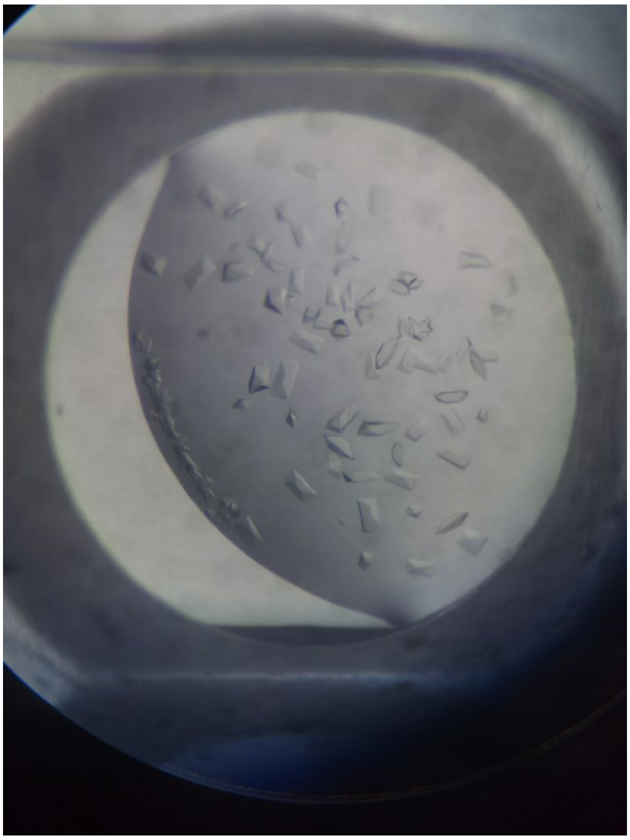

Figure S3. Micrograph of HvCHS crystals in the crystallization experiment. Multiple orthorhombic crystals grew in a clear, sitting drop containing 1µl protein (10mg/ml stock concentration) and 1µl reservoir solution (0.1 M MES/Imidazole pH6.5; 0.03 M $MgCl_2$, 0.03 M $CaCl_2$; 16% (v/v) glycerol, 8% (v/v) PEG4000) in 1-2 days at 4°C.

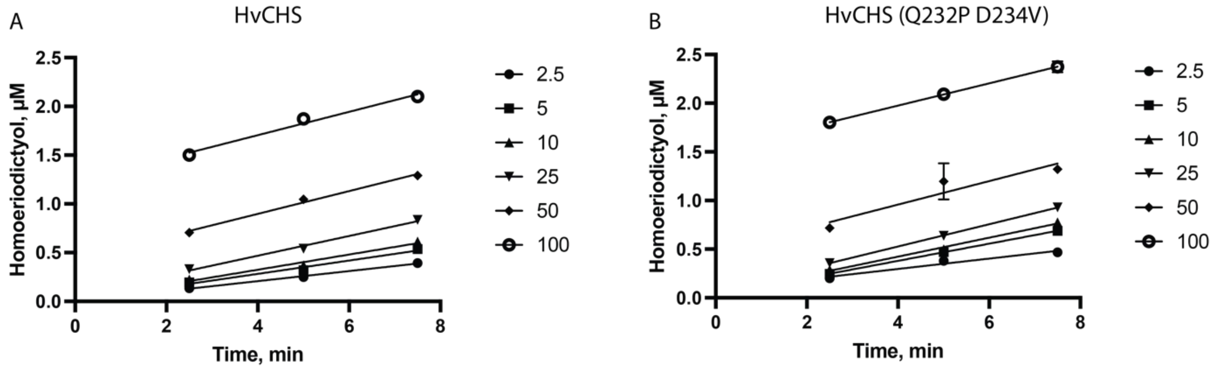

Figure S4. Time progress curves underlying the steady-state kinetics analysis. Concentration of homoeriodictyol obtained in *in vitro* turnovers catalyzed by A) HvCHS wild type and B) HvCHS double mutant in the presence of varying concentrations of feruloyl-CoA (2.5, 5, 10, 25, 50, and 100 µM) and a fixed concentration of malonyl-CoA (300 µM). Data points represent mean +/- SD, n=3, line represents linear regression to determine the apparent initial velocities for each substrate concentration.

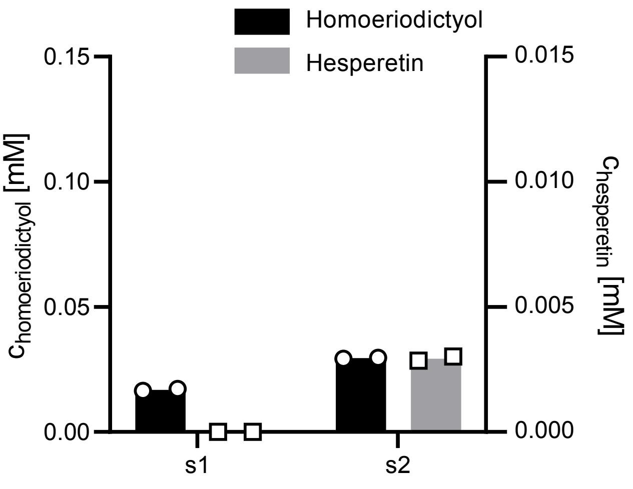

Figure S5. Flavonoid titers after fermentation with strains expressing either PhCHS from *Petunia hybrida* and 4CL from *Petroselinum crispum* (s1) or CHS from *Hordeum vulgare* and 4CL from *Oryza sativa* (s2). Small scale fermentation with 1 mM ferulic acid, and isoferulic acid added as precursors. Samples were taken after 32 h fermentation and analyzed by HPLC-MS. Black bar, left axis – mean of homoeriodictyol titer; grey bar, right axis – mean of hesperetin titer. Each experiment was duplicated.

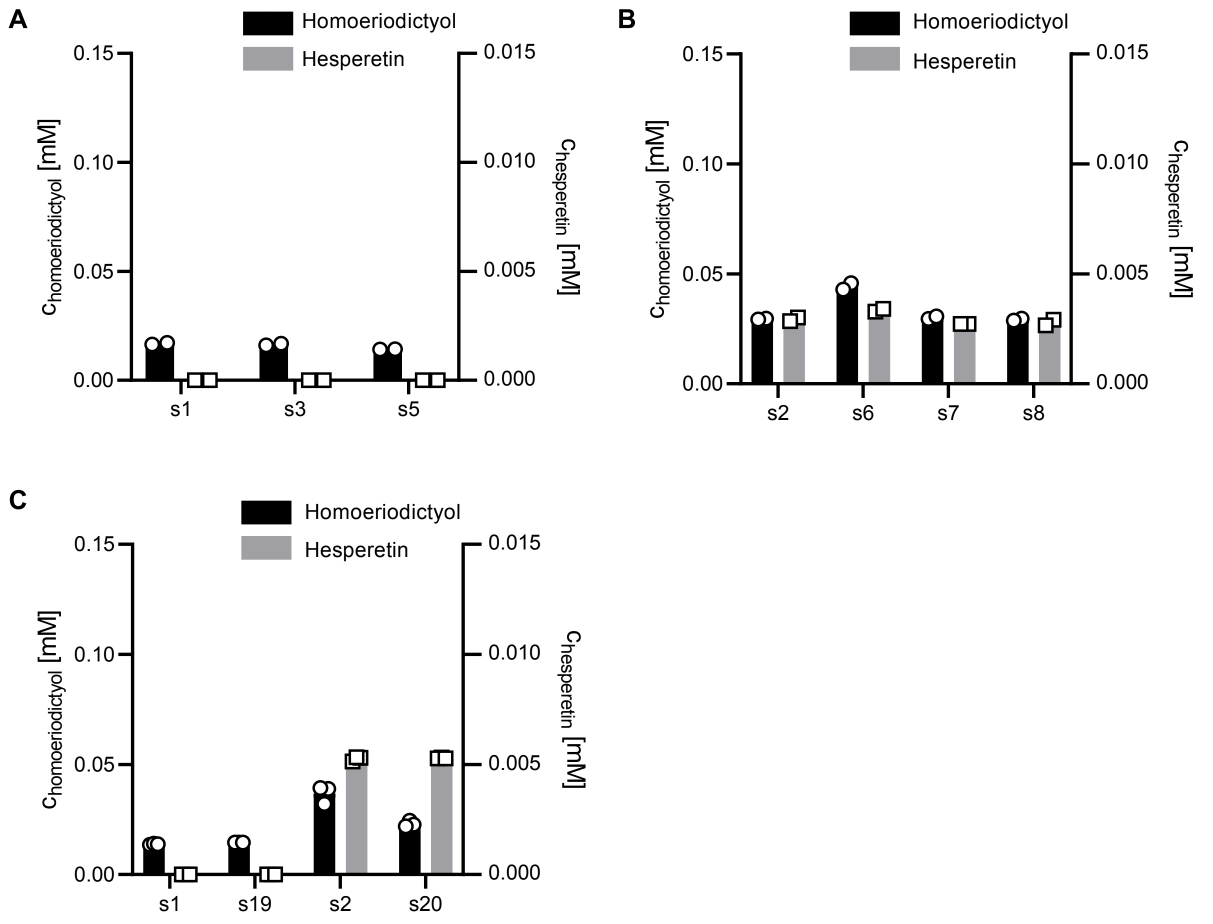

Figure S6. Flavonoid titers after fermentation with strains expressing different 4CL variants. A) Pc4CL variants (s1 Pc4CL wildtype, s3 Pc4CL del V342 variant, and s5 Pc4CL Q214A variant; bars represent mean of biological replicates, n=2.) B) Os4CL variants (s2 Os4CL wildtype, s6 Os4CL del V340 variant, s7 Os4CL Q212A variant, and s8 Os4CL S242A variant; bars represent mean of biological replicates, n=2. C) Double and triple mutant variants (s1 Pc4CL wildtype, s19 Pc4CL (Q214A, del V342) variant, s2 Os4CL wildtype, and s20 Os4CL (Q212A, S242A, del V340) variant); bars represent mean +/- SD of biological triplicates, n=3).

```
                                                                           |
HvCHS       -MAAVRLKEVRMAQRAEGLATVLAIGTAVPANCVYQATYPDYYFRVTKSEHLADLKEKFQ   59
OsCHS       MAAAVTVEEVRRAQRAEGPATVLAIGTATPANCVYQADYPDYYFRITKSEHMVELKEKFK   60
FhCHS       ---MVNVEEIRKAQRAEGPAAILAIGTATPPNAIEQSEYPDYYFRVTNSEDKVELKEKFK   57
PhCHS       ---MVTVEEYRKAQRAEGPATVMAIGTATPSNCVDQSTYPDFYFRITNSEHKTDLKEKFK   57
GmCHS       ---MVSVEEIRKAQRAEGPATVMAIGTATPPNCVDQSTYPDYYFRITNSEHMTELKEKFK   57
MsCHS       ---MVSVSEIRKAQRAEGPATILAIGTANPANCVEQSTYPDFYFKITNSEHKTELKEKFQ   57
                * :.* * ****** *:::***** * *.: *: ***:**::*:**. .:*****:

            ||  |
HvCHS       RMCDKSMIRKRHMHLTEEILIKNPKICAHMETSLDARHAIALVEVPKLGQGAAEKAIKEW   119
OsCHS       RMCDKSQIRKRYMHLTEEILQENPNMCAYMAPSLDARQDIVVVEVPKLGKAAAQKAIKEW   120
FhCHS       RMCEKSMIKKRYLYLTEDILKENPNVCAYMATSLDARQDMVVVEVPKLGKEAATRAIKEW   117
PhCHS       RMCEKSMIKKRYMHLTEEILKENPSMCEYMAPSLDARQDIVVVEVPKLGKEAAQKAIKEW   117
GmCHS       RMCDKSMIKKRYMYLNEEILKENPSVCAYMAPSLDARQDMVVVEVPKLGKEAATKAIKEW   117
MsCHS       RMCDKSMIKRRYMYLTEEILKENPNVCEYMAPSLDARQDMVVVEVPRLGKEAAVKAIKEW   117
            ***:** *::*:::*.*:** :**.:* :*  *****: :.:****:**: ** :*****

                         |
HvCHS       GQPLSKITHLVFCTTSGVDMPGADYQLTKLLGLSPTVKRLMMYQQGCFGGATVLRLAKDI   179
OsCHS       GQPRSRITHLVFCTTSGVDMPGADYQLAKMLGLRPNVSRLMMYQQGCFAGGTVLRVAKDL   180
FhCHS       GQPKSKITHLVFCTTSGVDMPGADYQLTKLLGLRPSVKRLMMYQQGCFAGGTVLRLAKDL   177
PhCHS       GQPKSKITHLVFCTTSGVDMPGCDYQLTKLLGLRPSVKRLMMYQQGCFAGGTVLRLAKDL   177
GmCHS       GQPKSKITHLIFCTTSGVDMPGADYQLTKLLGLRPSVKRYMMYQQGCFAGGTVLRLAKDL   177
MsCHS       GQPKSKITHLIVCTTSGVDMPGADYQLTKLLGLRPYVKRYMMYQQGCFAGGTVLRLAKDL   177
            *** *:****:.**********.****:*:*** * *.* ********.*.****:***:

                       |||  |             |     |||             * *
HvCHS       AENNRGARVLVVCSEITAMAFRGPCKSHLDSLVGHALFGDGAAAAIIGADPDQLDEQPVF   239
OsCHS       AENNRGARVLAVCSEITAVTFRGPSESHLDSMVGQALFGDGAAAVIVGSDPDEAVERPLF   240
FhCHS       AENNRGARVLVVCSEITAVTFRGPSESHLDSLVGQALFGDGAAALIVGSDAIEGIERPIF   237
PhCHS       AENNKGARVLVVCSEITAVTFRGPNDTHLDSLVGQALFGDGAGAIIIGSDPIPGVERPLF   237
GmCHS       AENNKGARVLVVCSEITAVTFRGPTDTHLDSLVGQALFGDGAAAVIVGSDPLP-VEKPLF   236
MsCHS       AENNKGARVLVVCSEVTAVTFRGPSDTHLDSLVGQALFGDGAAALIVGSDPVPEIEKPIF   237
            ****:*****.****:**::****  .:****:**:*******.* *:*:*    *:*:*

                         |||      ||||  |    |
HvCHS       QLVSASQTILPESEGAIDGHLTEAGLTIHLLKDVPGLISENIEQALEDAFEPLGIHNWNS   299
OsCHS       QMVSASQTILPDSEGAIDGHLREVGLTFHLLKDVPGLISKNIERALGDAFTPLGISDWNS   300
FhCHS       EMVSAAQTILPDSEGAIDGHLREVGLTFHLLKDVPGIISKNIEKSLEEAFKPLGITDYNS   297
PhCHS       ELVSAAQTLLPDSHGAIDGHLREVGLTFHLLKDVPGLISKNIEKSLEEAFKPLGISDWNS   297
GmCHS       QLVWTAQTILPDSEGAIDGHLREVGLTFHLLKDVPGLISKNIEKALVEAFQPLGISDYNS   296
MsCHS       EMVWTAQTIAPDSEGAIDGHLREAGLTFHLLKDVPGIVSKNITKALVEAFEPLGISDYNS   297
            ::* ::**: *:*.******* *.***:********::*:** ::* :** **** ::**

                ||||                             |
HvCHS       IFWIAHPGGPAILDRVEDRVGLDKKRMRASREVLSEYGNMSSASVLFVLDVMRKSSAKDG   359
OsCHS       IFWVAHPGGPAILDQVEAKVGLDKERMRATRHVLSEYGNMSSACVLFILDEMRKRSAEDG   360
FhCHS       LFWIAHPGGPAILDQVEAKIGLKPEKLRATRHVLSEYGNMSSACVLFILEEMRKKSAEEK   357
PhCHS       LFWIAHPGGPAILDQVEIKLGLKPEKLKATRNVLSNYGNMSSACVLFILDEMRKASAKEG   357
GmCHS       IFWIAHPGGPAILDQVEAKLGLKPEKMEATRHVLSEYGNMSSACVLFILDQMRKKSIENG   356
MsCHS       IFWIAHPGGPAILDQVEQKLALKPEKMNATREVLSEYGNMSSACVLFILDEMRKKSTQNG   357
            :**:**********:** ::.*. :::.*:*.***:*******.***:*: *** * ::

                             |
HvCHS       LATTGEGKDWGVLFGFGPGLTVETLVLHSVPVPVPTAASA   399
OsCHS       HATTGEGMDWGVLFGFGPGLTVETVVLHSVPITAGAAA--   398
FhCHS       NGTTGEGLEWGVLFGFGPGLTVETVVLHSVEA--------   389
PhCHS       LGTTGEGLEWGVLFGFGPGLTVETVVLHSVAT--------   389
GmCHS       LGTTGEGLDWGVLFGFGPGLTVETVVLRSVTL--------   388
MsCHS       LKTTGEGLEWGVLFGFGPGLTIETVVLRSVAI--------   389
              ***** :************:**:**:**
```

Figure S7. Multiple sequence alignments of those proteins that are structurally most similar to HvCHS as identified by Dali search against the PDB. Gray box: catalytic cysteine; pink bars: flavonoid binding residues, black bars: malonyl-CoA binding residues, purple box: active site residues that were mutated in this study; blue box: surface loop residues that were mutated in this study with stars marking the most beneficial mutations; color coding within black box according to CLUSTAL W color coding.

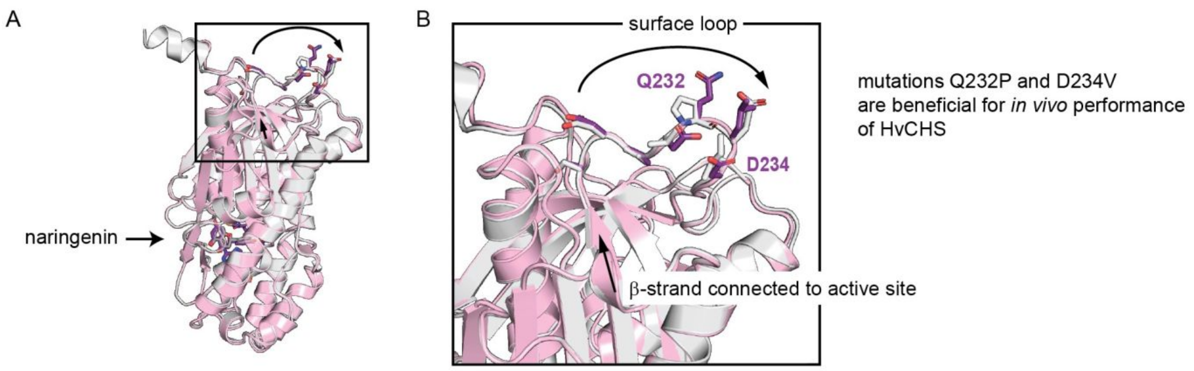

Figure S8. Illustration of surface loop in HvCHS (PDB: 8B35, pink) compared to MsCHS (PDB: 1CGK, grey). A) Overview of the structure. B) Magnification of the area in the box. The Q232P and D234V single and double point mutations in this loop appear to modulate HvCHS expression level and affinity to ferulic acid and are therefore beneficial for the *in vitro* and *in vivo* performance of the enzyme. Atom coloring in stick representation: oxygen=red, nitrogen=blue, phosphorous=orange, carbon=color matches the cartoon color of the corresponding structure. Compared to Fig. 2A, the model is rotated by 180° along the y-axis.
